# Endoscopic observation of different repair patterns in human traumatic tympanic membrane perforations^☆^

**DOI:** 10.1016/j.bjorl.2017.06.011

**Published:** 2017-08-03

**Authors:** Peng Huang, Shujun Zhang, Xinhong Gong, Xuesong Wang, Zi-Han Lou

**Affiliations:** a Binzhou Centre Hospital, Department of Otorhinolary, Shandong, China; b Binzhou Centre Hospital, Department of Physiatry, Shandong, China; c Xinxiang Medical University, Department of Clinical Medicine, Henan, China

**Keywords:** Tympanic membrane perforation, Trauma, Epithelial cell, Granulation tissue, Endoscope, Perfuração da membrana timpânica, Trauma, Célula epitelial, Tecido de granulação, Endoscópio

## Abstract

**Introduction:**

In the last decade, there has been an increasing use of biomaterial patches in the regeneration of traumatic tympanic membrane perforations. The major advantages of biomaterial patches are to provisionally restore the physiological function of the middle ear, thereby immediately improving ear symptoms, and act as a scaffold for epithelium migration. However, whether there are additional biological effects on eardrum regeneration is unclear for biological material patching in the clinic.

**Objective:**

This study evaluated the healing response for different repair patterns in human traumatic tympanic membrane perforations by endoscopic observation.

**Methods:**

In total, 114 patients with traumatic tympanic membrane perforations were allocated sequentially to two groups: the spontaneous healing group (*n* = 57) and Gelfoam patch-treated group (*n* = 57). The closure rate, closure time, and rate of otorrhea were compared between the groups at 3 months.

**Results:**

Ultimately, 107 patients were analyzed in the two groups (52 patients in the spontaneous healing group vs. 55 patients in the Gelfoam patch-treated group). The overall closure rate at the end of the 3 month follow-up period was 90.4% in the spontaneous healing group and 94.5% in the Gelfoam patch-treated group; the difference was not statistically significant (*p* > 0.05). However, the total average closure time was significantly different between the two groups (26.8 ± 9.1 days in the spontaneous healing group vs. 14.7 ± 9.1 days in the Gelfoam patch-treated group, *p* < 0.01). In addition, the closure rate was not significantly different between the spontaneous healing group and Gelfoam patch-treated group regardless of the perforation size. The closure time in the Gelfoam patch-treated group was significantly shorter than that in the spontaneous healing group regardless of the perforation size (small perforations: 7.1 ± 1.6 days vs. 12.6 ± 3.9, medium-sized perforations: 13.3 ± 2.2 days vs. 21.8 ± 4.2 days, and large perforations: 21.2 ± 4.7 days vs. 38.4 ± 5.7 days; *p* < 0.01).

**Conclusion:**

In the regeneration of traumatic tympanic membrane perforations, Gelfoam patching not only plays a scaffolding role for epithelial migration, it also promotes edema and hyperplasia of granulation tissue at the edges of the perforation and accelerates eardrum healing.

## Resumo

**Introdução:**

Na última década, houve um uso crescente de placas biomateriais na regeneração de perfurações traumáticas da membrana timpânica. As principais vantagens das placas de biomateriais são restaurar provisoriamente a função fisiológica da orelha média, assim melhoram imediatamente os sintomas da orelha e atuam como um suporte para a migração do epitélio. No entanto, não se sabe se há efeitos clínicos adicionais na regeneração do tímpano em relação ao fragmento de material biológico.

**Objetivo:**

Avaliar a resposta de cicatrização para diferentes padrões de reparo em perfurações de membrana timpânica traumáticas humanas por meio de observação endoscópica.

**Método:**

Foram alocados 114 pacientes com perfurações de membrana timpânica traumáticas sequencialmente para dois grupos: o de cicatrização espontânea (n = 57) e o tratado com esponja de Gelfoam (n = 57). A velocidade de fechamento, o tempo de fechamento e a taxa de otorreia foram comparados entre os grupos aos três meses.

**Resultados:**

Foram analisados 107 pacientes nos dois grupos (52 no de cicatrização espontânea e 55 no tratado com esponja de Gelfoam). A velocidade global de fechamento no final do período de seguimento de três meses foi de 90,4% no grupo de cicatrização espontânea e de 94,5% no grupo tratado com esponja de Gelfoam; a diferença não foi estatisticamente significativa (*p* > 0,05). No entanto, o tempo total médio de fechamento foi significativamente diferente entre os dois grupos (26,8 ± 9,1 dias no de cicatrização espontânea versus 14,7 ± 9,1 dias no tratado com esponja de Gelfoam, *p* < 0,01). Além disso, a velocidade de fechamento não foi significativamente diferente entre o grupo de cicatrização espontânea e o grupo tratado com esponja de Gelfoam, independentemente do tamanho da perfuração. O tempo de fechamento no grupo tratado com esponjas de Gelfoam foi significativamente menor do que no grupo de cicatrização espontânea, independentemente do tamanho da perfuração (pequenas perfurações: 7,1 ± 1,6 dias vs. 12,6 ± 3,9, perfurações de tamanho médio: 13,3 ± 2,2 dias vs. 21,8 ± 4,2 dias e grandes perfurações: 21,2 ± 4,7 dias vs. 38,4 ± 5,7 dias; *p* < 0,01).

**Conclusão:**

Na regeneração de PMT traumáticas, a esponja de Gelfoam não só desempenha um papel de estrutura à migração epitelial, mas também promove edema e hiperplasia de tecido de granulação nas bordas da perfuração e acelera a cicatrização do tímpano.

## Introduction

Traumatic tympanic membrane perforations (TMPs) tend to heal spontaneously; the healing time for most TMPs is 1–3 months. Many studies have shown that a biological patch could shorten the closure time of traumatic TMPs and even improve the closure rate.^1^, ^2^, ^3^, ^4^, ^5^, ^6^, ^7^, ^8^, ^9^, ^10^ Previous authors reported that a key feature of biological patches was to provide a scaffold for epithelial cell migration to aid the repair process of traumatic TMPs. However, previous studies were based on experimental histologic examinations.^4^, ^7^, ^8^, ^11^, ^12^ Whether there are additional biological effects on eardrum regeneration is unclear for biological material patching in the clinic. The objective of this study was to observe the repair process for different repair patterns (spontaneous healing vs. Gelfoam patching) in human traumatic TMPs through dynamic endoscopic observation from a morphologic viewpoint.

## Patients and methods

### Subjects

A prospective, sequential allocation and controlled clinical study was conducted at Otorhinolaryngology, Head and Neck Surgery department. The protocol of study had been approved by our Ethics of Research Committee (n° 20141201). The investigators had obtained written consent from each participant or their guardians. It included 107 patients with traumatic TMP, from January 2015 and June 2016.

### Inclusion criteria

Cases that met the following inclusion criteria were analyzed: (i) a slap- or fist-induced TMP within 7 days of the injuries; (ii) age ≥16 years and (iii) dry TMPs.

### Exclusion criteria

(i) A wet or moist TMP with bloody, watery, and/or purulent otorrhea at the first hospital visit; (ii) severe vertigo or ossicular disruption suspected upon physical examination or imaging; (iii) blast injury, water sport injury, or direct penetrating injury; and (iv) a history of previous middle ear disease, atrophic eardrum, or myringosclerosis.

The tympanic membrane was examined using an endoscope after removing cerumen or blood clots from the external auditory canal (EAC) using a cotton bud soaked in povidone-iodine solution. The tympanic membrane was simultaneously photographed using a digital video camera, and the size of the perforation was analyzed using Image J software (NIH, Bethesda, MD, USA). Each perforation was assigned to one of three categories based on the affected portion of the eardrum: small < 1/8; medium 1/8 to 1/4; and large > 1/4.^9^ Age, sex, date of injury, presence or absence of otorrhea, and associated clinical findings, including hearing loss, vertigo, and tinnitus, were recorded at each visit. Since perforation healing is associated with successful closure of the air-bone gap, audiometric examination was not performed in this study.^5^, ^6^, ^13^

### Treatment allocation

The 114 subjects were allocated into two groups: spontaneous healing (*n* = 57) and Gelfoam patching (*n* = 57). This was performed by the principal investigator with the help of a registered nurse using a sequential allocation method. Specifically, consecutive subjects who both met the inclusion criteria and signed the consent form were alternately allocated to the two groups based on the order of their initial hospital visit, perforation size, and date of returning the signed consent form.

## Treatments

### Spontaneous healing group

Patients in this group received no intervention but underwent regular follow-up.

### Gelfoam patch-treated group

The external ear canal was cleaned with a cotton bud soaked in a povidone-iodine solution. None of the perforation edges underwent trimming. A modified and pressed Gelfoam sheet, larger than the perforation, was soaked in 0.5% chlortetracycline ointment and then placed onto the tympanic membrane remnant (i.e., onlay technique), completely covering the perforated area so that at least 2 mm of the Gelfoam patch overlapped the margin.

### Follow-up

Oral amoxicillin was given to all subjects for 1 week. Follow-up was scheduled twice a week following the initiation of treatment. Thereafter, follow-up was scheduled once a week until complete closure of the perforation was achieved, or for up to 3 months. The tympanic membrane was examined repeatedly by endoscopy at all follow-up visits. The initial Gelfoam patch was removed and a fresh piece of Gelfoam was placed onto the tympanic membrane at each visit in the Gelfoam group. To reduce clinician bias, clinical events such as tympanic membrane closure or the presence of otorrhea were photo-documented using color slides. If a patient had severe vertigo, signs of perilymph leakage were evaluated and the patient was excluded from the study. Perforation closure was confirmed by endoscopic examination. Demographic data and outcome measures were expressed as the mean ± SD and analyzed using a paired Chi-Squared test or *t*-test with SPSS software (ver. 11.0 for Windows; SPSS Inc., Chicago, IL, USA). Differences were considered statistically significant at *p* < 0.05.

## Results

### Patient demographics

In total, 114 cases met the inclusion criteria and were analyzed. Of these cases, loss of follow-up occurred in four patients in the spontaneous healing group and two patients in the Gelfoam patch-treated group. In addition, one middle ear infection was seen in the spontaneous healing group; however, no middle ear infections were seen in the Gelfoam patch-treated group. Thus, 107 patients were ultimately analyzed in the two groups (52 in the spontaneous healing group vs. 55 in the Gelfoam patch-treated group). Of the 52 patients in the spontaneous healing group, the perforation size was small in 14 patients, medium in 21 patients, and large in 17 patients. Of the 55 patients in the Gelfoam patch-treated group, the perforation size was small in 12 patients, medium in 24 patients, and large in 19 patients. In large TMP, folded edge was seen in 12 patients in spontaneous healing group while in 16 patents in the Gelfoam patch-treated group. The demographic data for the patients in the two groups are presented in Table 1. The average age, male-to-female patient ratio, size of the perforation, size of the ear, folded edge, and average elapsed time between injury and the hospital visit were similar in the two groups (*p* > 0.05).

**Table 1:** Demographic characteristic of spontaneous healing and Gelfoam patching group.

Group	Spontaneous healing	Gelfoam patching	*p* value
No.	52	55	–
Age (Y)	36.4 ± 5.2	37.1 ± 4.8	0.486^a^
Sex (M:F)	11:41	17:38	0.472^b^
Size (S:M:L)	14:21:17	12:24:19	0.516^b^
Duration (days)	3.7 ± 2.1	3.2 ± 1.8	0.553^a^
Side of ear (L:R)	46:6	47:8	0.847^b^
Folded edge (with:without)	12:5	16:3	0.378^b^

*p* < 0.05 was considered statistically significant.

a *t* test.

b *χ*^2^ test.

### Healing outcome

The patients were followed for a total of 3 months or until complete closure of the perforation. The healing outcome is summarized in Table 2. The overall closure rate at the end of the 3 month follow-up period was 90.4% in the spontaneous healing group and 94.5% in the Gelfoam patch-treated group; the difference was not statistically significant (*p* > 0.05). However, the total average closure time was significantly different between the two groups (26.8 ± 9.1 days in the spontaneous healing group vs. 14.7 ± 9.1 days in the Gelfoam patch-treated group; *p* < 0.01).

**Table 2:** Healing outcome of different size perforations of spontaneous healing and Gelfoam patching group.

Group	Perforation size	No	Closure rate (%)	Average closure time
Spontaneous healing	Small-sized	14	13 (92.85)	12.6 ± 3.9
	Medium-sized	21	21 (100.4)	21.8 ± 4.2
	Large-sized	17	13 (76.5)	38.4 ± 5.7

Gelfoam patch	Small-sized	12	12 (100.0)	7.1 ± 1.6
	Medium-sized	24	23 (95.8)	13.3 ± 2.2
	Large-sized	19	17 (89.5)	21.2 ± 4.7

The healing outcome based on the perforation size classification after 3 months of follow-up is shown in Table 2. The closure rate was not significantly different between the spontaneous healing group and the Gelfoam patch-treated group regardless of the perforation size. However, the closure time in the Gelfoam patch-treated group was significantly shorter than that in the spontaneous healing group regardless of the perforation size (small perforations: 7.1 ± 1.6 days vs. 12.6 ± 3.9; medium-sized perforations: 13.3 ± 2.2 days vs. 21.8 ± 4.2 days, and large perforations: 21.2 ± 4.7 days vs. 38.4 ± 5.7 days; *p* < 0.01).

### Endoscopic observation

**Figure 1:**
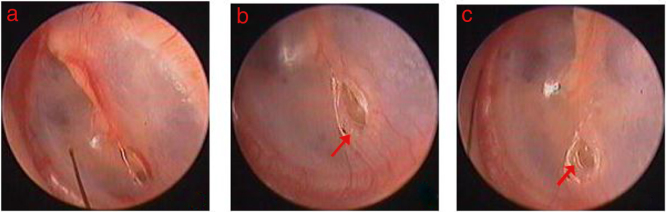
Spontaneous healing process of small perforation: (A–C) was the 1 day, 5 days and 8 days following perforation. Red indicated epithelium proliferation at the edge.

In the 52 patients in the spontaneous healing group, hyperemia and edema at the perforation edges were seen within 48 h, and various degrees of proliferation of the thin and transparent epithelium occurred and formed a few areas of regenerated eardrum at 3–4 days. Edema at the perforation edges decreased gradually, and turbidity occurred immediately following regeneration of the eardrum at 4–5 days. Thereafter, the epithelium accumulated gradually and migrated toward the center of the perforation at the edge. Seven small-sized perforations achieved complete closure within 1 week. The epithelium continued to grow and migrated toward the center of the perforation at the edges of unhealed perforations until they were completely closed (Fig. 1). However, the increasing epithelium caused an abnormality in the center of the perforation and outward migration in one small-sized perforation and two large-sized perforations. The outwardly migrating epithelium gradually formed a crust and did not close the perforation within 3 months. The epithelium did not continue to grow after about 6 weeks to 2 months and failed to close within 3 months in two large-sized perforations. In addition, turbidity of the regenerated eardrum did not occur, and only the atrophic regenerated eardrum closed the perforation in a small-sized perforation. The folded edges gradually became necrosis and formed curst over time, eventually migrated to EAC after perforation closure and did not affect the healing process in 11 large perforations with folded edge. Of the 47 healed TMPs, the morphology of the regenerated eardrum was normal in 46 patients, and the regenerated eardrum was atrophic in one small-sized perforation.

**Figure 2:**
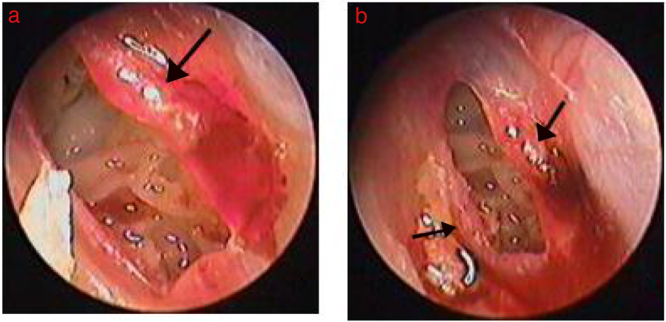
The healing process of perforation after Gelfoam patching: (A) 3 days after Gelfoam patching treatment; (B) 4 days after Gelfoam patching treatment. Black arrows indicate granulation tissue, edema, and exudate at the margin.

**Figure 3:**
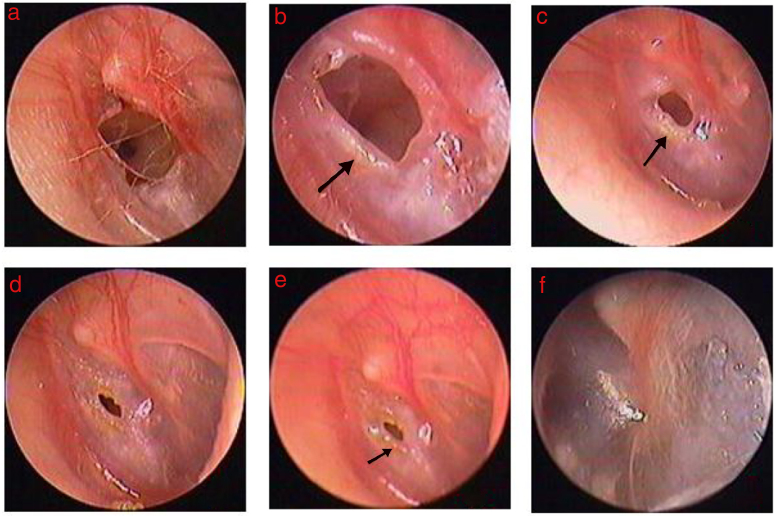
The healing process of perforation after Gelfoam patching: (A) 1st following perforation; (B–E) 2, 4, 7, and 9 days, and 2 weeks after Gelfoam patching. Black arrows indicate granulation tissue, edema, and exudate at the margin.

Among the 55 patients in the Gelfoam patch-treated group, edema, reddish granular hyperplastic tissue, and exudation was seen at the perforation edges within 2–3 days after Gelfoam patching in 48 patients. The regenerated tissue was thick, reddish granular tissue that increased gradually and migrated toward the center of the perforation. Subsequently, epithelization followed on the surface of the granular tissue, and finally the reddish granular tissue closed the perforation. Significant edema and hyperplasia of the granular tissue occurred and gradually became dominant at the edges in four large-sized perforations. Four large-sized perforations completely closed at 9–12 days (Figure 2, Figure 3). The folded edges gradually became edema and dissolved, the proliferation of red granulation tissue or proliferous eardrum was seen and gradually increased over time in 14 large perforations with folded edge. However, edema, reddish granular hyperplastic tissue, and exudation were not seen during the follow-up period in one small-sized perforation and two large-sized perforations, and these three TMPs failed to close within 3 months. Of the 52 healed eardrums in the Gelfoam patch-treated group, the healed eardrum was thicker than the uninjured eardrum, but the thickness of the healed eardrum became normal about 3–7 days after healing.

## Discussion

There are at least two advantages to biological material patching in the regeneration of traumatic TMPs^1^, ^2^, ^3^, ^5^, ^6^, ^9^, ^10^: (1) a biological material patch of similar thickness to the eardrum covers the perforation and can provisionally restore the physiological function of the middle ear, thereby immediately improving ear symptoms (e.g., tinnitus and ear fullness); and (2) a biological material patch acts as a scaffold for epithelium migration and shortens the closure time of traumatic TMPs. This study also suggests that the closure time of the Gelfoam patch-treated group was significantly shorter than that of the spontaneous healing group regardless of the perforation size. However, the therapeutic effect of a biological material patch on traumatic TMPs is not completely understood.

Most scholars believe that a biological material patch acts only as a scaffold for epithelium migration and does not improve the closure rate.^2^, ^5^, ^6^ In our study, the spontaneous healing process of human traumatic TMPs was similar to that in the experimental group; that is, the perforations were first closed by proliferation of the epidermal layer of the drum from proliferation centers, followed by proliferation of the fibrous layer.^14^, ^15^, ^16^ The centripetal migration of a single thin and transparent layer (most likely a proliferating epithelial layer) was the first event seen by endoscopy, and thickening of this layer (presumably due to emergence and growth of the fibrous and mucosal layers underneath) followed in most traumatic TMPs. Nevertheless, outward migration of a single thin epidermal layer occurred at 1–2 weeks, gradually formed the crust, and ultimately failed to close the perforation in a few patients. In addition, the epithelium did not continue to grow and turbidity of the regenerated eardrum did not occur after about 6 weeks to 2 months. In addition, closure failed to occur within 3 months in two large-sized perforations. Thus, epithelial cells may deviate from the center of a perforation and migrate outward during spontaneous healing, or hyperplastic epithelial cells may not be sufficient to repair the perforation. However, in the Gelfoam patch-treated group, the outward migration and deviation of regenerated tissue at the perforation edge was not seen in all patients. Edema, reddish granular hyperplastic tissue, and exudation were seen at the perforation edges after Gelfoam patching. Reddish granular tissue migrated centripetally and first closed the perforation, followed by epithelization in most cases. Interestingly, significant edema and hyperplasia of granulation tissue at the edges resulted in faster healing in four large-sized perforations; in others, edema and reddish granular hyperplastic tissue did not occur and closure failed. We speculate that the inflammatory response to a biological material patch at the perforation edge plays a vital role in the regeneration of traumatic TMPs.

The regeneration of TMPs is a complex biological process that involves epithelial cell proliferation and migration, fibroblast hyperplasia, and vascular tissue remodeling.^17^ Gelfoam patching promoted edema and granulation hyperplasia at perforation edges, and the granulation tissue was rich in neovascularization and fibroblasts, which provided the necessary oxygen and nutrition for wound healing,^18^ thereby accelerating TMP healing. A large amount of exudate at the edges not only helped avoid tissue necrosis and adhesion, the wet environment also stimulated the rapid growth of epithelial cells and fibroblasts, thereby facilitating wound healing.^19^, ^20^ Although chlortetracycline ointment was a confounding factor in this study, chlortetracycline ointment keeps the eardrum moist and promotes granulation tissue hyperplasia at perforation edges, thereby aiding eardrum healing.^21^, ^22^ However, the number of patients receiving chlortetracycline ointment in our study was small; the chlortetracycline ointment would dry within a few days, such that the effects on eardrum healing were negligible. In clinical studies, an antibiotic ointment is usually used to secure the patching material (e.g., hen egg Shell^2^ and Steri-Strips patch^5^) and prevent it from detaching from the eardrum. A clinical study of paper patching alone for traumatic TMPs reported a 92% closure rate.^10^

Previous studies overemphasized the scaffold function of patches and ignored the inflammatory response and granulation hyperplasia of biological materials at the edges. A histologic study demonstrated that different biological materials may cause varying degrees of inflammation.^4^, ^11^, ^12^ Clinical studies also found that the repair of traumatic TMPs was not completely dependent on the scaffold support; on the contrary, the topical application of certain agents alone (e.g., growth factors^23^, ^24^ and ofloxacin ear drops^25^) promoted faster healing compared to agents combined with biological materials. Similarly, some scholars overemphasized the role of proliferation and migration of the epithelium on eardrum healing and deemphasized the proliferation of granulation tissue in the fibrous layer at the edges. This and a previous histologic study found that the proliferation of granulation tissue in the fibrous layer plays an important role in the healing of traumatic TMPs.^26^ Without proliferation of the fibrous layer, atrophic healing of the eardrum and failure to heal could result. In our study, one perforation ultimately formed an atrophic eardrum in the spontaneous healing group, and the regenerated eardrum did not subsequently become turbid during spontaneous healing of the perforation. No reddish granular tissue occurred during the healing process, and closure failed to occur within 3 months in three patients in the Gelfoam patch-treated group. A few experimental studies also found that granulation tissue proliferation in the fibrous layer closed the perforation.^27^, ^28^

Our evaluation of the healing process of traumatic TMPs was based on the assessment of morphology by endoscopic observation. The absence of histologic evidence has obvious drawbacks; however, it is impossible to obtain this evidence for ethical reasons. Single granulation tissue first closed the perforation; thus, the synchronous proliferation of granulation tissue in the fibrous and epithelial layers in the Gelfoam patch-treated group should be studied further. In addition, perforations with an atrophic eardrum and myringosclerosis were excluded from this study; the therapeutic effect of Gelfoam patching on these patients requires further study.

## Conclusions

In the regeneration of traumatic TMPs, Gelfoam patching not only plays a scaffolding role for epithelial migration, it also promotes edema and hyperplasia of granulation tissue at the edges and accelerates eardrum healing. Thus, patching should be considered for large-sized perforations; however, traumatic TMPs have an excellent capacity for spontaneous healing. Thus, spontaneous healing should be recommended first for small- and medium-sized perforations. In addition, we found that perforations healed more rapidly when edema and hyperplasia of granulation tissue at the edges became more significant. Thus, it is important that a clinic seek biological materials that are non-ototoxic, produce no local pain, and cause a strong inflammatory reaction in the future.

## Funding

This study was supported by the Science and Technology Agency of Zhejiang Province, Health & Medicine Agency of Zhejiang Province, and Science and Technology Agency of Yiwu, China (Grants n° 2013C33176, 2015KYB420, and 2015-3-06).

## Conflicts of interest

The authors declare no conflicts of interest.
